# Definition and management of arrhythmia-induced cardiomyopathy: findings from the European Heart Rhythm Association survey

**DOI:** 10.1093/europace/euae112

**Published:** 2024-05-02

**Authors:** Teodor Serban, Patrick Badertscher, Jeanne du Fay de Lavallaz, Rui Providencia, Federico Migliore, Giacomo Mugnai, Diego Penela, Laura Perrotta, Michael Kühne, Christian Sticherling, Kyoung-Ryul Julian Chun

**Affiliations:** Department of Cardiology, University Hospital Basel, University of Basel, Petersgraben 4, 4031 Basel, Switzerland; Cardiovascular Research Institute Basel, University Hospital Basel, University of Basel, Spitalstrasse 2, 4056 Basel, Switzerland; Department of Cardiology, University Hospital Basel, University of Basel, Petersgraben 4, 4031 Basel, Switzerland; Cardiovascular Research Institute Basel, University Hospital Basel, University of Basel, Spitalstrasse 2, 4056 Basel, Switzerland; Department of Cardiology, University Hospital Basel, University of Basel, Petersgraben 4, 4031 Basel, Switzerland; Cardiovascular Research Institute Basel, University Hospital Basel, University of Basel, Spitalstrasse 2, 4056 Basel, Switzerland; Department of Cardiology, St Bartholomew’s Hospital, London, UK; Department of Cardiac, Thoracic and Vascular Sciences and Public Health, University of Padova, Padova, Italy; Department of Cardiology, University Hospital of Verona, Verona, Italy; Department of Cardiology, Medical Centre Teknon, Grupo Quironsalud, Barcelona, Spain; Department of Cardiology, Careggi University Hospital, Florence, Italy; Department of Cardiology, University Hospital Basel, University of Basel, Petersgraben 4, 4031 Basel, Switzerland; Cardiovascular Research Institute Basel, University Hospital Basel, University of Basel, Spitalstrasse 2, 4056 Basel, Switzerland; Department of Cardiology, University Hospital Basel, University of Basel, Petersgraben 4, 4031 Basel, Switzerland; Cardiovascular Research Institute Basel, University Hospital Basel, University of Basel, Spitalstrasse 2, 4056 Basel, Switzerland; Department of Electrophysiology, Cardiology and Angiology Center Bethanien, Frankfurt, Germany

**Keywords:** Arrhythmia-induced cardiomyopathy, Pacemaker, Heart failure, Atrial fibrillation, Premature ventricular contractions, Antwerp score

## Abstract

**Aims:**

Arrhythmia-induced cardiomyopathy (AiCM) represents a subtype of acute heart failure (HF) in the context of sustained arrhythmia. Clear definitions and management recommendations for AiCM are lacking. The European Heart Rhythm Association Scientific Initiatives Committee (EHRA SIC) conducted a survey to explore the current definitions and management of patients with AiCM among European and non-European electrophysiologists.

**Methods and results:**

A 25-item online questionnaire was developed and distributed among EP specialists on the EHRA SIC website and on social media between 4 September and 5 October 2023. Of the 206 respondents, 16% were female and 61% were between 30 and 49 years old. Most of the respondents were EP specialists (81%) working at university hospitals (47%). While most participants (67%) agreed that AiCM should be defined as a left ventricular ejection fraction (LVEF) impairment after new onset of an arrhythmia, only 35% identified a specific LVEF drop to diagnose AiCM with a wide range of values (5–20% LVEF drop). Most respondents considered all available therapies: catheter ablation (93%), electrical cardioversion (83%), antiarrhythmic drugs (76%), and adjuvant HF treatment (76%). A total of 83% of respondents indicated that adjuvant HF treatment should be started at first HF diagnosis prior to antiarrhythmic treatment, and 84% agreed it should be stopped within six months after LVEF normalization. Responses for the optimal time point for the first LVEF reassessment during follow-up varied markedly (1 day–6 months after antiarrhythmic treatment).

**Conclusion:**

This EHRA Survey reveals varying practices regarding AiCM among physicians, highlighting a lack of consensus and heterogenous care of these patients.

## Graphical Abstract

**Graphical Abstract:**
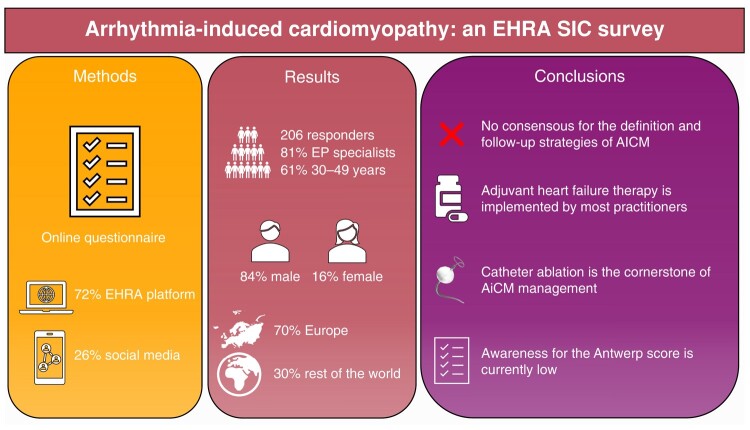

**What’s new?**

- Despite being a well-known cause of HF, arrhythmia-induced cardiomyopathy (AiCM) is often underdiagnosed and is an important cause of potentially reversible LVEF depression in patients with new-onset arrhythmias.
- No unanimous definition of AiCM was identified, but most experts agree that it should be defined as a systolic HF in the context of an arrhythmia. There was a high degree of uncertainty regarding the threshold of LVEF drop/improvement qualifying for the definition of AiCM.
- The management of AiCM as reported by the respondents is complex and involves the use of all available therapies.
- Follow-up strategies for AiCM differ greatly between practitioners highlighting the clinical need for a consensus.

## Introduction

Arrhythmia-induced cardiomyopathy (AiCM) is an increasingly recognized entity characterized by new cardiac dysfunction in the context of an often persistent cardiac arrhythmia, which is potentially reversible upon its effective control.^1^ The potential reversibility of AiCM provides a compelling reason for early diagnosis and intervention, which usually lead to significant improvement in cardiac function and patient outcomes.^2,3^

The exact pathophysiological mechanisms underlying AiCM are incompletely understood, but it is believed that arrhythmia-induced myocardial remodelling plays a central role.^1,4,5^ This remodelling can lead to adverse structural and functional changes, which may be at least partially reversed with the normalization of heart rate and/or rhythm control.^6–8^ The clinical presentation of AiCM is heterogeneous, ranging from asymptomatic drop in left ventricular ejection fraction (LVEF) to decompensated acute heart failure (HF).^5^

Diagnosis of AiCM is challenging due to the absence of specific diagnostic criteria and the broad differential diagnosis of other HF aetiologies, especially when first manifesting as dilated cardiomyopathy.^9^ Traditionally, AiCM has been diagnosed retrospectively after observing an improvement in ventricular function following treatment of the arrhythmia.^10^ However, the potential reversibility of the pathology warrants proactive diagnosis particularly with the advent of more sophisticated imaging techniques and biomarkers and effective therapies such as catheter ablation (CA).^11–15^ Recently, a novel predictive model attempted an early identification of LVEF recovery after CA for atrial fibrillation (AF).^16,17^

## Aim

This European Heart Rhythm Association Scientific Committee (EHRA SIC) questionnaire aimed to elucidate the definitions and management of AiCM among European and non-European electrophysiologists.

## Methods

### Online questionnaire

The questionnaire was developed by the EHRA Scientific Initiatives Committee. The survey electronic link was sent to ∼4000 members of EHRA and EHRA Young EP between 4 September 2023 and 5 October 2023, and also promoted via social media. The online-based questionnaire consisted of single- and multiple-choice questions assessing how AiCM is currently defined, the risk factors for AiCM depending on the arrhythmia underlying AiCM [i.e. atrial fibrillation- or premature ventricular contraction (PVC)-induced CM], type and duration of follow-up and monitoring, the clinical and imaging parameters for identification potential AiCM patients, and the treatment and adjuvant treatment of AiCM. The full questionnaire was approved by all investigators and is provided in the Supplementary material online, *Appendix*. The response was voluntary, anonymous, and GDPR compliant.

### Statistical analysis

Continuous variables are presented as mean ± standard deviation (SD) or median and interquartile range. Categorical variables are expressed as numbers and percentages. Test for normality of the distribution was assessed visually. All analyses were performed using R Studio (version 4.2.1, Vienna, Austria).

## Results

**Figure 1:**
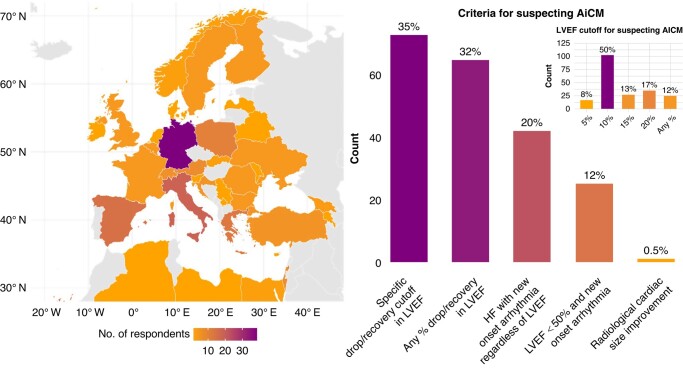
Left—distribution of the respondents in Europe (70% of the total respondents). Right—graph showing the preferred definition of arrhythmia-induced cardiomyopathy (AiCM), single-choice. Indented in the top right corner is the most preferred LVEF drop/improvement in the context of an arrhythmia to be used to trigger diagnosis of AiCM, also single-choice. AICM, arrhythmia-induced cardiomyopathy; LVEF, left ventricular ejection fraction.

From a total of 255 responses, 49 questionnaires were empty (contained no responses to any question) leaving 206 questionnaires that were analysed further. Of the 206 respondents, 32 (16%) were female and most respondents were between 40 and 49 (33%) years old, followed by the age group of 30–39 (28%) and 50–59 (23%). The vast majority was EP specialists (81%), followed by general cardiologists (11%), EP fellows (7%), and Internal Medicine specialists (2%). Most respondents worked at university hospitals (47%), followed by private hospitals (17%), public hospitals (16%), specialized public cardiology centres (16%), and 4% worked in private practices. Most respondents (47%) were practicing in Western Europe, followed by Eastern Europe (23%), Americas (11%), Asia Pacific regions (11%), and Africa and Middle East (8%). A total of 54 (26%) of the responses were gathered using social media, while the rest 152 (72%) were completed through the EHRA platform (*Figure 1*).

### Definition of arrhythmia-induced cardiomyopathy

When asked how the respondents defined AiCM, 73 (35%) defined it as a specific drop/recovery cut-off in LVEF after the occurrence/resolution of the arrhythmia, while 65 (32%) indicated that any % drop in LVEF in relationship with arrhythmia occurrence/treatment should prompt the diagnosis of AiCM. A total of 42 (20%) respondents indicated that a new onset of HF (i.e. either systolic or diastolic) should trigger the diagnosis of AiCM, while 25 (12%) indicated that a LVEF < 50% in the context of a new arrhythmia onset prompts the suspicion of AiCM.

When asked which specific LVEF cut-off should be chosen to define AiCM in the context arrhythmia onset/treatment, most respondents indicated a cut-off of 10% (102, 50%), followed by 20% (35, 17%), 15 (27, 13%), and 5% (17, 8%), while 25 (25, 12%) indicated that any % LVEF drop should trigger the suspicion of AICM (*Figure 1*).

### Arrhythmias related to arrhythmia-induced cardiomyopathy

Regarding the arrhythmias that most common cause AiCM, AF was chosen by the majority of respondents (190, 92%), followed by high amounts of right-ventricular pacing (133, 65%), atrial flutter (127, 62%), and PVCs (124, 60%). Permanent junctional reciprocating tachycardia was chosen by 76 (37%) of the respondents, atrioventricular nodal re-entry tachycardia in 12%, and ventricular tachycardia by only 3 (1.5%) respondents.

### Differential diagnosis in arrhythmia-induced cardiomyopathy

The respondents were asked whether further evaluation for other types of HF with cardiac magnetic resonance (CMR), coronary angiography, or serial brain natriuretic peptide (BNP) measurements is warranted for differential diagnosis of AiCM. Cardiac magnetic resonance was chosen by 24 (16%) respondents, while coronary angiography was chosen by 28 (14%) respondents and 100 (49%) respondents use serial BNP measurements.

**Figure 2:**
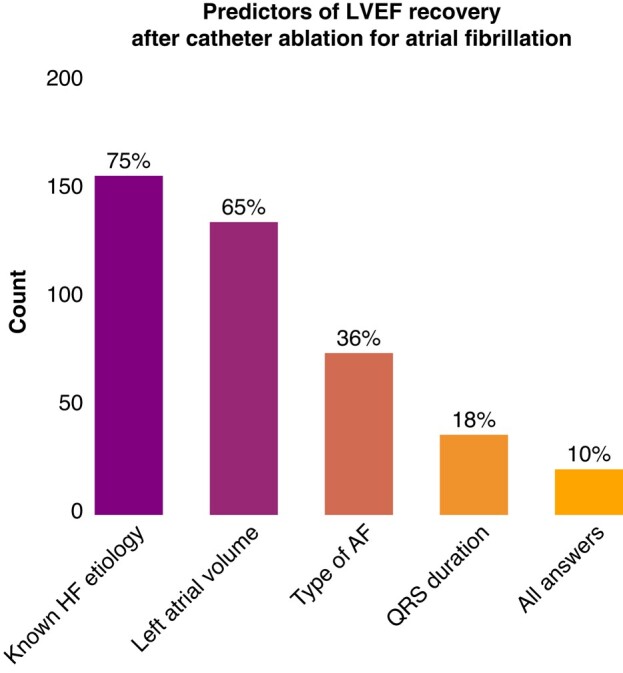
Distribution of the answers regarding the putative predictors of left ventricular ejection fraction recovery after catheter ablation for atrial fibrillation (multiple choice). Only 10% of the respondents chose all answers (i.e. the Antwerp score). AF, atrial fibrillation; HF, heart failure; LVEF, left ventricular ejection fraction.

The respondents were further questioned regarding the known predictors for AiCM (i.e. Antwerp Score, multiple choice): 155 (75%) mentioned the lack of a known HF cause, 134 (65%) chose left atrial volume, 74 (36%) chose type of AF, and 37 (18%) chose QRS duration. Only 21 (10%) of the respondents chose all four answers (*Figure 2*).

### Antiarrhythmic treatment in arrhythmia-induced cardiomyopathy

**Figure 3:**
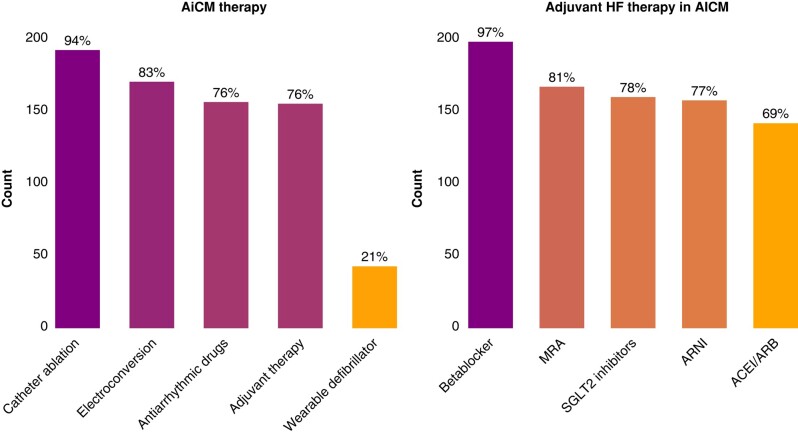
Left—distribution of the responses regarding the therapy of arrhythmia-induced cardiomyopathy. Multiple choices were allowed. Right—distribution of the responses regarding adjuvant heart failure therapy in AICM. Multiple choices were allowed. AICM, arrhythmia-induced cardiomyopathy; ACE, angiotensin conversion enzyme antagonist; ARB, angiotensin receptor blocker; ARNI, angiotensin receptor/neprilysin inhibitor; MRA, mineralocorticoid receptor antagonist.

When questioned about the therapy options for AiCM (multiple choice), most respondents considered all rhythm control strategies—193 (94%) CA, 171 (83%) electrical cardioversions, as well as 157 (76%) antiarrhythmic drugs. A wearable defibrillator was chosen by 43 (21%, *Figure 3*).

### Adjuvant treatment in arrhythmia-induced cardiomyopathy

Most respondents mentioned that they regularly recommend adjuvant HF therapy in AiCM patients (156, 76%). When asked to define adjuvant HF treatment in AiCM (multiple choice), the majority included drug treatment with betablockers—(193, 94%) and/or mineralocorticoid receptor antagonists—(167, 81%), sodium glucose transport protein 2 (SGLT2) antagonists—(161, 78%), angiotensin receptor–neprilysin inhibitor—(158, 77%), and angiotensin converting enzyme inhibitors or receptor blockers—(142, 69%, *Figure 3*). Sixty-two (30%) of respondents chose 4 medications, 22 (11%) 3 medications, and 14 (7%) 2 medications.

Most respondents (170, 83%) reported that the adjuvant drug therapy should be started at the time of HF diagnosis, before rhythm control. In contrast, 36 (17%) respondents chose to start the drug therapy only in case of persistent HF after rhythm control.

Most respondents (173, 84%) stopped the adjuvant drug therapy usually within 6 months after HF normalization, while 32 (16%) opted for lifelong treatment. Half of the respondents reported that the follow-up of AiCM patients after LVEF recovery may be ended within the first year—53 (26%) at one year, 30 (15%) at 6 months, and 24 (12%) at 3 months, while the other half of the respondents—98 (48%) usually follow their AiCM patients for over a year. A mean of 10% (±10) of the AiCM patients will receive loop recorders to monitor for early arrhythmia recurrence after restoration of sinus rhythm.

### Premature ventricular contractions-induced cardiomyopathy

**Figure 4:**
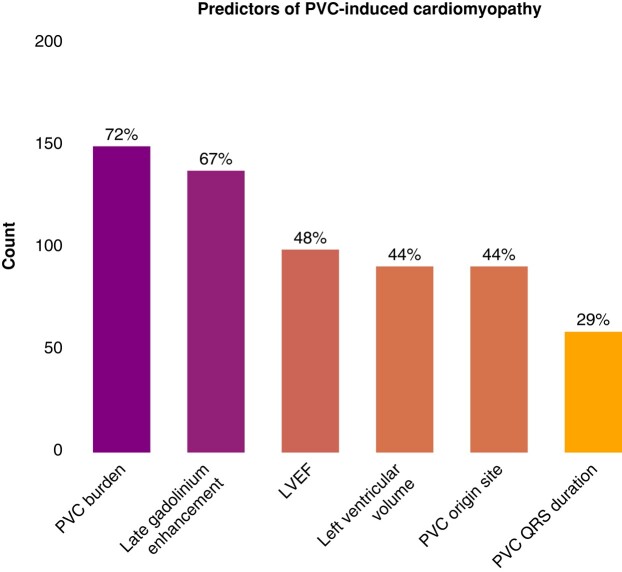
Distribution of the answers regarding the known predictors of premature ventricular contraction-induced cardiomyopathy. LVEF, left ventricular ejection fraction; PVC, premature ventricular contractions.

When questioned about predictors of risk factors for PVC-induced CM, 149 (72%) chose the overall PVC burden—with a median PVC burden cut-off of 11% (±7), 137 (67%) chose late gadolinium enhancement (LGE) on cardiac MRI examination, 99 (48%) chose LVEF at presentation, 91 (44%) chose the left ventricular volume at presentation, 90 (44%) the site and origin of the PVCs, and 59 (29%) chose the QRS duration of the PVC. There was uncertainty regarding the assessment of right ventricular (RV) function in addition to LV function in PVC-induced CM patients: 85 (41%) respondents chose to assess the RV function, 74 (36%) respondents assess the RV function in some patients, and 46 (22%) of the respondents do not routinely assess the RV function (*Figure 4*).

## Discussion

The purpose of this EHRA SIC survey was to offer a snapshot of current definitions used for AiCM, its risk factors, predictors, therapeutic possibilities, and follow-up strategies. We report that there is a lack of a universally accepted definition for AiCM, with a high degree of uncertainty regarding a specific LVEF drop/recovery cut-off in the context of a new onset/treatment of the culprit arrhythmia.

The questionnaire offered several other important insights: (1) AF is the most commonly identified arrhythmia causing AiCM, followed by high amounts of ventricular pacing, atrial flutter, and PVCs; (2) there is inconsistent use of MRI or coronary angiography for differential diagnosis, with a surprising high reliance on serial BNP measurements; (3) antiarrhythmic treatments, particularly CA, are widely used, alongside adjunctive HF therapy initiated at the time of HF diagnosis. However, one in five respondent chose to start a medical therapy only in case of persistent HF after rhythm control. The time point of stopping adjunctive HF medication is unclear. (4) Follow-up practices after LVEF recovery vary between practitioners, showing an even split between short-term (<1year) and long-term monitoring (>1 year) for arrhythmia recurrence; (5) PVC burden is recognized as a key risk factor in PVC-induced cardiomyopathy; and 6) there is a great variability in the assessment of right ventricular function in PVC-induced cardiomyopathy.

The survey clearly identifies AF as the leading arrhythmia associated with AiCM, followed by other supraventricular and ventricular arrhythmias. This aligns with existing literature emphasizing the high prevalence of AF in AiCM cases.^18,19^

In terms of differential diagnosis, the low endorsement for advanced imaging techniques such as CMR and coronary angiography points to a potential underutilization of these modalities. This is rather surprising, since the CAMERA-MRI^20^ showed that restoration of sinus rhythm via CA results in significant improvement in ventricular function, particularly in patients with absent ventricular fibrosis on CMR. The reliance on serial BNP measurements reflects a non-invasive, cost-effective approach, but may miss structural heart disease that could be elucidated by imaging There are only limited data available regarding a biomarker-driven approach. Nia *et al*.^21^ assessed 40 patients with supraventricular tachycardia and HF with serial NT-proBNP measurements at baseline, Day 1, and weekly over a period of 4 weeks.

Arrhythmia-induced cardiomyopathy patients revealed higher decrement velocity of NT-proBNP compared to patients with other causes of HF.

The Antwerp score has been recently proposed to identify AICM patients from a population with depressed LVEF undergoing CA for AF showing variable predictive power in different populations.^16,17,22^ According to the current questionnaire, as only a very small number 21 (10%) of the respondents chose all four proposed parameters as predictors for LVEF recovery after CA for AF, we report that the awareness for this predictive model is currently very low. This tool, however, requires prospective validation.

The consensus on antiarrhythmic treatment strategies, particularly the widespread use of CA,^23,24^ suggests a strong preference for rhythm control in AiCM, aligning with recent data^19,25–28^ and current guidelines recommending CA as a first-line treatment when AiCM is highly probable independent of symptom status (class I, level of evidence B, ESC Guidelines).^29,30^

The results also highlight the importance of adjunctive HF therapy, with a notable consensus on initiating treatment at first HF diagnosis irrespective of the relationship with the arrhythmia. However, no clear time point as to whether/when this adjuvant therapy should be ended after LVEF recovery was identified. This ambiguity may have various reasons. One of them might be the increasing evidence, that over time, cardiac arrhythmias can lead to progressive ventricular remodelling via tachycardia and irregular ventricular rhythm.^9,31^ While LVEF can normalize, left ventricular diameter stay dilated and diffuse fibrosis as detected by CMR might persist as a late outcome after AiCM and thus might argue for lifelong HF therapy.^32^

The follow-up practices after antiarrhythmic treatment of AICM showed once again a split. There was no consensus with regard to the best time point for LVEF reassessment after antiarrhythmic treatment and with half of the respondents ending the follow-up of AICM patients within a year post-LVEF recovery and the other half extending beyond a year. This divergence underscores the lack of standardized follow-up duration and calls for research to optimize monitoring periods.^16,17,22,33,34^

For PVC-induced cardiomyopathy, the identification of PVC burden as a primary risk factor echoes current understanding,^35^ yet the survey indicates variability in the assessment of right ventricular function, suggesting an area where guidelines could provide clarity.^36^

There is a lack of guidance from the ESC guidelines regarding AiCM, and there is no clear algorithm for diagnosis or management. In this context, some responses such as the time to LVEF reassessment might indicate a level IIb indication, while other answers clearly underlie the importance of further research, such as studies evaluating the role of ongoing HF therapy after rhythm restoration.

As limitations of this survey, we report that most respondents came from Western Europe and that we did not include pacemaker-induced cardiomyopathy into our questionnaire.

## Conclusion

This EHRA Survey reveals varying practices in defining and managing AiCM among European and non-European physicians. There is an unmet need for a consensus in the definition and management of AiCM patients to improve patient care.

## Supplementary Material

euae112_Supplementary_Data

## Data Availability

All relevant data are within the manuscript and its Supporting Information files.

## References

[1] Simantirakis EN, Koutalas EP, Vardas PE. Arrhythmia-induced cardiomyopathies: the riddle of the chicken and the egg still unanswered? Europace 2012;14:466–73. 22084300 10.1093/europace/eur348

[2] González-Ferrero T, Bergonti M, López-Canoa JN, Arias FG, Eiras Penas S, Spera F et al Atrial fibrillation ablation in patients with arrhythmia-induced cardiomyopathy: a prospective multicentre study. ESC Heart Fail 2023;10:3055–66. 37593841 10.1002/ehf2.14448 PMC10567669

[3] Hékimian G, Paulo N, Waintraub X, Bréchot N, Schmidt M, Lebreton G et al Arrhythmia-induced cardiomyopathy: a potentially reversible cause of refractory cardiogenic shock requiring venoarterial extracorporeal membrane oxygenation. Heart Rhythm 2021;18:1106–12. 33722763 10.1016/j.hrthm.2021.03.014

[4] Raymond-Paquin A, Nattel S, Wakili R, Tadros R. Mechanisms and clinical significance of arrhythmia-induced cardiomyopathy. Canad J Cardiol 2018;34:1449–60. 30404750 10.1016/j.cjca.2018.07.475

[5] Huizar JF, Ellenbogen KA, Tan AY, Kaszala K. Arrhythmia-induced cardiomyopathy. J Am Coll Cardiol 2019;73:2328–44. 31072578 10.1016/j.jacc.2019.02.045 PMC6538508

[6] Kuck K-H, Lebedev DS, Mikhaylov EN, Romanov A, Gellér L, Kalējs O et al Catheter ablation or medical therapy to delay progression of atrial fibrillation: the randomized controlled atrial fibrillation progression trial (ATTEST). Europace 2021;23:362–369a. 33330909 10.1093/europace/euaa298 PMC7947582

[7] Blomström-Lundqvist C, Naccarelli G V, McKindley DS, Bigot G, Wieloch M, Hohnloser SH. Effect of dronedarone vs. placebo on atrial fibrillation progression: a *post hoc* analysis from ATHENA trial. Europace 2023;25:845–54. 36758013 10.1093/europace/euad023 PMC10062319

[8] Koldenhof T, Wijtvliet PEPJ, Pluymaekers NAHA, Rienstra M, Folkeringa RJ, Bronzwaer P et al Rate control drugs differ in the prevention of progression of atrial fibrillation. Europace 2022;24:384–9. 34414430 10.1093/europace/euab191 PMC8892061

[9] Serban T, de Lavallaz J dF, Mannhart D, Pfister O, van der Stouwe JG, Kaufmann BA et al Echocardiographic pattern of left ventricular function recovery in tachycardia-induced cardiomyopathy patients. ESC Heart Fail 2023;10:2386–94. 37218391 10.1002/ehf2.14365 PMC10375182

[10] Lishmanov A, Chockalingam P, Senthilkumar A, Chockalingam A. Tachycardia-induced cardiomyopathy: evaluation and therapeutic options. Congest Heart Fail 2010;16:122–6. 20557332 10.1111/j.1751-7133.2010.00147.x

[11] Fenelon G, Wijns W, Andries E, Brugada P. Tachycardiomyopathy: mechanisms and clinical implications. Pacing Clin Electrophysiol 1996;19:95–106. 8848384 10.1111/j.1540-8159.1996.tb04796.x

[12] Kuniss M, Pavlovic N, Velagic V, Hermida JS, Healey S, Arena G et al Cryoballoon ablation vs. antiarrhythmic drugs: first-line therapy for patients with paroxysmal atrial fibrillation. Europace 2021;23:1033–41. 33728429 10.1093/europace/euab029 PMC8286851

[13] Andrade JG, Chierchia G-B, Kuniss M, Wazni OM. New evidence: cryoballoon ablation vs. antiarrhythmic drugs for first-line therapy of atrial fibrillation. Europace 2022;24:ii14–21. 35661868 10.1093/europace/euab246

[14] Schnabel RB, Marinelli EA, Arbelo E, Boriani G, Boveda S, Buckley CM et al Early diagnosis and better rhythm management to improve outcomes in patients with atrial fibrillation: the 8th AFNET/EHRA consensus conference. Europace 2023;25:6–27. 35894842 10.1093/europace/euac062 PMC9907557

[15] Arbelo E, Dagres N. The 2020 ESC atrial fibrillation guidelines for atrial fibrillation catheter ablation, CABANA, and EAST. Europace 2022;24:ii3–7. 35661865 10.1093/europace/euab332

[16] Bergonti M, Spera F, Tijskens M, Bonomi A, Saenen J, Huybrechts W et al A new prediction model for left ventricular systolic function recovery after catheter ablation of atrial fibrillation in patients with heart failure: the ANTWOORD study. Int J Cardiol 2022;358:45–50. 35443194 10.1016/j.ijcard.2022.04.040

[17] Serban T, du Fay de Lavallaz J, Barker DC, Sticherling C, Kühne M, Badertscher P. Validation of a novel score to predict which patients with atrial fibrillation and depressed left ventricular ejection fraction will respond to catheter ablation. Rev Esp Cardiol (Engl Ed) 2023;76:745–8. 37085115 10.1016/j.rec.2023.03.014

[18] Brachmann J, Sohns C, Andresen D, Siebels J, Sehner S, Boersma L et al Atrial fibrillation burden and clinical outcomes in heart failure. JACC Clin Electrophysiol 2021;7:594–603. 33640355 10.1016/j.jacep.2020.11.021

[19] Marrouche NF, Brachmann J, Andresen D, Siebels J, Boersma L, Jordaens L et al Catheter ablation for atrial fibrillation with heart failure. N Engl J Med 2018;378:417–27. 29385358 10.1056/NEJMoa1707855

[20] Prabhu S, Taylor AJ, Costello BT, Kaye DM, McLellan AJA, Voskoboinik A et al Catheter ablation versus medical rate control in atrial fibrillation and systolic dysfunction: the CAMERA-MRI study. J Am Coll Cardiol 2017;70:1949–61. 28855115 10.1016/j.jacc.2017.08.041

[21] Nia AM, Gassanov N, Dahlem KM, Caglayan E, Hellmich M, Erdmann E et al Diagnostic accuracy of NT-proBNP ratio (BNP-R) for early diagnosis of tachycardia-mediated cardiomyopathy: a pilot study. Clin Res Cardiol 2011;100:887–96. 21538234 10.1007/s00392-011-0319-y

[22] Bergonti M, Ascione C, Marcon L, Pambrun T, Della Rocca DG, Ferrero TG et al Left ventricular functional recovery after atrial fibrillation catheter ablation in heart failure: a prediction model. Eur Heart J 2023;44:3327–35. 37387689 10.1093/eurheartj/ehad428

[23] Kautzner J, Neuzil P, Lambert H, Peichl P, Petru J, Cihak R et al EFFICAS II: optimization of catheter contact force improves outcome of pulmonary vein isolation for paroxysmal atrial fibrillation. Europace 2015;17:1229–35. 26041872 10.1093/europace/euv057 PMC4535556

[24] Del Monte A, Almorad A, Pannone L, Della Rocca DG, Bisignani A, Monaco C et al Pulmonary vein isolation with the radiofrequency balloon catheter: a single centre prospective study. Europace 2023;25:896–904. 36738245 10.1093/europace/euad017 PMC10062286

[25] Sohns C, Fox H, Marrouche NF, Crijns HJGM, Costard-Jaeckle A, Bergau L et al Catheter ablation in end-stage heart failure with atrial fibrillation. N Engl J Med 2023;389:1380–9. 37634135 10.1056/NEJMoa2306037

[26] Hsu L-F, Jaïs P, Sanders P, Garrigue S, Hocini M, Sacher F et al Catheter ablation for atrial fibrillation in congestive heart failure. N Engl J Med 2004;351:2373–83. 15575053 10.1056/NEJMoa041018

[27] Prabhu S, Taylor AJ, Costello BT, Kaye DM, McLellan AJA, Voskoboinik A et al Catheter ablation versus medical rate control in atrial fibrillation and systolic dysfunction. J Am Coll Cardiol 2017;70:1949–61. 28855115 10.1016/j.jacc.2017.08.041

[28] Kawaji T, Shizuta S, Aizawa T, Yamagami S, Kato M, Yokomatsu T et al Impact of catheter ablation for atrial fibrillation on cardiac disorders in patients with coexisting heart failure. ESC Heart Fail 2021;8:670–9. 33305495 10.1002/ehf2.13160 PMC7835577

[29] Hindricks G, Potpara T, Dagres N, Arbelo E, Bax JJ, Blomström-Lundqvist C et al 2020 ESC guidelines for the diagnosis and management of atrial fibrillation developed in collaboration with the European Association for Cardio-Thoracic Surgery (EACTS). Eur Heart J 2021;42:373–498. 32860505 10.1093/eurheartj/ehaa612

[30] Joglar JA, Chung MK, Armbruster AL, Benjamin EJ, Chyou JY, Cronin EM et al 2023 ACC/AHA/ACCP/HRS guideline for the diagnosis and management of atrial fibrillation: a report of the American College of Cardiology/American Heart Association joint committee on clinical practice guidelines. Circulation 2024;149:e1–156. 38033089 10.1161/CIR.0000000000001193 PMC11095842

[31] Dandamudi G, Rampurwala AY, Mahenthiran J, Miller JM, Das MK. Persistent left ventricular dilatation in tachycardia-induced cardiomyopathy patients after appropriate treatment and normalization of ejection fraction. Heart Rhythm 2008;5:1111–4. 18675220 10.1016/j.hrthm.2008.04.023

[32] Ling L, Kalman JM, Ellims AH, Iles LM, Medi C, Sherratt C et al Diffuse ventricular fibrosis is a late outcome of tachycardia-mediated cardiomyopathy after successful ablation. Circ Arrhythm Electrophysiol 2013;6:697–704. 23884195 10.1161/CIRCEP.113.000681

[33] Han FT, Kiser R, Nixon JV, Wood MA, Ellenbogen KA. What is the time course of reversal of tachycardia-induced cardiomyopathy? Europace 2011;13:139–41. 20682551 10.1093/europace/euq298

[34] Hennings E, Aeschbacher S, Coslovsky M, Paladini RE, Meyre PB, Voellmin G et al Association of bone morphogenetic protein 10 and recurrent atrial fibrillation after catheter ablation. Europace 2023;25:euad149. 37314197 10.1093/europace/euad149 PMC10265951

[35] du Fay de Lavallaz J, Mézier J, Mertz L, Mannhart D, Serban T, Knecht S et al Risk factors for the development of premature ventricular complex-induced cardiomyopathy: a systematic review and meta-analysis. J Interv Card Electrophysiol 2022;66:1145–63. 36414810 10.1007/s10840-022-01421-8 PMC10333144

[36] Fink T, Sciacca V, Sommer P. Catheter ablation of premature ventricular contractions with multiple morphologies in tachycardia-induced cardiomyopathy: all or nothing? Europace 2023; 25:euad064. 10.1093/europace/euad064 PMC10227652 36942441

