# Supplementary material for: Definition and management of arrhythmia-induced cardiomyopathy: findings from the European Heart Rhythm Association survey
Source: Europace. 2024 May 2;26(5):euae112. doi: 10.1093/europace/euae112 (PMC11094751; doi:10.1093/europace/euae112)
Supplement: euae112_Supplementary_Data [file euae112_supplementary_data.docx]

**Management of Arrhythmia-induced Cardiomyopathy**

Dear Doctor,

We are conducting a European Heart Rhythm Association (EHRA) survey to gather information about arrhythmia-induced cardiomyopathy (AICM) and would like to invite you to participate. The survey will take only 5 minutes of your time and will provide valuable insights into current practices, challenges and perceptions of this condition. Your input will be critical in shaping the future of care for patients with AICM.

We understand that your time is valuable and appreciate your willingness to contribute to this important research. Your participation in this 5-minute survey is completely voluntary and all responses will be kept confidential. Your responses will make a significant impact on the future of AICM care.

Thank you in advance for your time and contributions.

Best regards,

EHRA Scientific Committee

1. **GDPR Disclaimer**

- Your participation is anonymous
- We will not disclose your identity to any third party.
- We comply with the European General Data Protection Regulation (GDPR) 2016/679. Any personal data processed in connection with this survey will be treated confidentially and only used by the ESC for the purposes of market research and not for promotion. Survey results will be kept for a maximum of 48 months for analysis and quality control purposes. We take all reasonable care to prevent any unauthorized access to your personal data. We respect your privacy and your right to access, modify, or remove your personal data. At any time, you can ask to know what personal data is being held. If you have any questions about data protection or require further information, please contact our data protection officer (DPO) at dpo@escardio.org.
- You have the right to end your participation in this survey at any time.
- **Have you read the above and agree to participate in this survey?**

**YES**

**NO**

1. **Abbreviations:**

- **Afib:** Atrial fibrillation
- **AICM:** Arrhythmia-induced Cardiomyopathy
- **CM:** Cardiomyopathy
- **CMR:** cardiac magnetic resonance imaging
- **CRT:** Cardiac resynchronisation therapy
- **EHRA:** European Heart Rhythm Association
- **EP:** Electrophysiology
- **ESC:** European Society of Cardiology
- **GDPR:** General data protection regulation
- **HF:** Heart failure
- **LVEF:** Left ventricular ejection fraction
- **PM:** Pacemaker
- **PVC:** premature ventricular contraction
- **TTE:** transthoracic echocardiography examination

1. **General questions:**
2. Please indicate your age.

*a. Slider 20-100 years*

1. Please indicate your gender
2. Female
3. Male
4. In which country do you practice?
5. EHRA country list
6. Primary working environment
7. University Hospital
8. Specialised public cardiology centre
9. Public general/district/community hospital
10. Private hospital/clinic
11. Private practice
12. Other, please specify
13. Current working position
14. Cardiologist – EP specialist
15. Cardiologist
16. EP fellow
17. Cardiology fellow
18. General practitioner
19. Internal medicine resident
20. Other
21. What criteria are you using to diagnose arrhythmia-induced cardiomyopathy (AICM)?
22. A specific drop/recovery cut-off in left ventricular ejection fraction (LVEF) after the occurrence/resolution of the arrhythmia
23. Any % drop/recovery in LVEF after the occurrence/resolution of the arrhythmia
24. LVEF < 50% and new onset arrhythmia prompts the supposition of AICM
25. Regardless of LVEF, any kind of heart failure (HF) in relationship with a new onset arrhythmia prompts the supposition of AICM
26. Other, please specify
27. If a specific cut-off for drop/recovery in LVEF after the occurrence/resolution of the arrhythmia is required for the diagnosis of AICM, the following cut-offs should be applied:
    1. 5%
    2. 10%
    3. 15%
    4. 20%
28. Which arrhythmia, in your opinion, is most prone to develop AICM?
    1. Atrial fibrillation
    2. Atrial flutter
    3. Premature Ventricular Contractions (PVC)
    4. High amount of right ventricular pacing burden
    5. Supraventricular tachycardia such as atrioventricular nodal reentry tachycardia (AVNRT) or permanent junctional reciprocating tachycardia (PJRT)
    6. Other (please specify)
29. Magnetic resonance imaging (CMR) is warranted in all patients with ongoing arrhythmia and reduced LVEF?
    1. No
    2. In some patients
    3. In all patients
30. Coronary angiography or CT coronary angiogram is warranted in all patients with ongoing arrhythmia and reduced LVEF?
    1. No
    2. In some patients
    3. In all patients
31. After restoration of sinus rhythm, LVEF assessment should be performed
    1. > 1 day
    2. ≥ 1 week
    3. ≥ 1 month
    4. ≥ 3 months
    5. ≥ 6 months
    6. Other (please specifiy)
32. Serial measurements of biomarkers such as NT-proBNP after rhythm control are useful to distinguish AICM from other HF entities?
    1. Yes
    2. No
33. How do you treat AICM? (Select all that apply)
    1. Catheter ablation
    2. Antiarrhythmic drug treatment
    3. Electrical cardioversion (if applicable)
    4. Adjuvant HF therapy according to LVEF
    5. Wearable Defibrillator in specific circumstances
    6. Other (please specify)
34. Adjuvant/ Adjunctive HF therapy in AICM consists of? (Select all that apply)
    1. Betablocker
    2. Angiotensin-converting enzyme (ACE) inhibitor
    3. Angiotensin receptor/neprilysin inhibitor
    4. Mineralocorticoid receptor antagonist
    5. Sodium-glucose cotransporter 2 (SGLT2) inhibitor
    6. Other (please specify)
35. If you use adjuvant HF therapy for AICM, at what point do you initiate the therapy?
    1. At the time of the first HF diagnosis, before the invasive antiarrhythmic treatment in the case of systolic HF
    2. After the antiarrhythmic (medical/ non-medical) treatment
    3. After the antiarrhythmic treatment, only if there is persistent systolic HF (after > 1 week)
    4. Other (please specify):
36. If you use adjuvant HF therapy for AICM, at what point do you terminate the therapy in case of complete resolution of HF (
    1. Right after the normalisation of HF
    2. 1 month after the normalisation of HF
    3. 1-3 months after normalisation of HF
    4. 3-6 months after normalisation of HF
    5. Lifetime treatment
    6. Other (please specify):
37. What is your mode of follow-up in patients with AICM and resolution of LVEF?
    1. 3 months
    2. 6 months
    3. 12 months
    4. > 12 months
38. Do you use an implantable loop recorder (ILR) in patients with AICM after sinus rhythm restoration to allow early detection of arrhythmia recurrence?
    1. No
    2. In some patients
    3. In all patients
39. Regarding PVC-induced cardiomyopathy (CM) – reversible HF is a concern with a PVC burden as high as
    1. 1-5%
    2. 5-10%
    3. 10-15%
    4. 15-20%
    5. > 20%
40. Which of the following factors are predictors of a complete LVEF recovery after PVC ablation in patients with a suspected PVC induced CM? (multiples choices are allowed)
    1. baseline PVC burden
    2. Site of origin (left vs right sided PVCs)
    3. QRS duration
    4. baseline LVEF
    5. presence of fibrosis in the CMR
    6. baseline left ventricular diameter/volume
41. Do you routinely assess right ventricular function in patients with PVC-induced CM?
42. No
43. In some patients
44. In all patients

-------------------------------------------

Dear colleagues,

Thank you very much for completing the survey.

Your input is very much appreciated and will provide us with invaluable insight into the management of Arrhythmia-induced Cardiomyopathy in European EP centres.

If you have any comments or questions regarding this survey, feel free to contact us at:

[Teodor.serban@usb.ch](mailto:Teodor.serban@usb.ch)

[Patrick.badertscher@usb.ch](mailto:Patrick.badertscher@usb.ch)
